# First shotgun metagenomics study of Juan de Fuca deep-sea sediments reveals distinct microbial communities above, within, between, and below sulfate methane transition zones

**DOI:** 10.3389/fmicb.2023.1241810

**Published:** 2023-11-20

**Authors:** Felix Metze, John Vollmers, Florian Lenk, Anne-Kristin Kaster

**Affiliations:** Institute for Biological Interfaces (IBG 5), Karlsruhe Institute of Technology, Hermann-von-Helmholtz Platz, Karlsruhe, Germany

**Keywords:** metagenome assembled genomes, optimized DNA extraction, deep biosphere, hydrothermal fluid, marine subsurface, bacteria, archaea

## Abstract

The marine deep subsurface is home to a vast microbial ecosystem, affecting biogeochemical cycles on a global scale. One of the better-studied deep biospheres is the Juan de Fuca (JdF) Ridge, where hydrothermal fluid introduces oxidants into the sediment from below, resulting in two sulfate methane transition zones (SMTZs). In this study, we present the first shotgun metagenomics study of unamplified DNA from sediment samples from different depths in this stratified environment. Bioinformatic analyses showed a shift from a heterotrophic, Chloroflexota-dominated community above the upper SMTZ to a chemolithoautotrophic Proteobacteria-dominated community below the secondary SMTZ. The reintroduction of sulfate likely enables respiration and boosts active cells that oxidize acetate, iron, and complex carbohydrates to degrade dead biomass in this low-abundance, low-diversity environment. In addition, analyses showed many proteins of unknown function as well as novel metagenome-assembled genomes (MAGs). The study provides new insights into microbial communities in this habitat, enabled by an improved DNA extraction protocol that allows a less biased view of taxonomic composition and metabolic activities, as well as uncovering novel taxa. Our approach presents the first successful attempt at unamplified shotgun sequencing samples from beyond 50 meters below the seafloor and opens new ways for capturing the true diversity and functional potential of deep-sea sediments.

## Introduction

Oceans cover more than two-thirds of the earth’s surface, and approximately 95% of the seabed is too deep to enable photosynthesis for primary biomass production (Jørgensen and Boetius, 2007). Nonetheless, microbes are metabolically active in those deep sediments and basement rocks with a proposed depth limit to life of 10,000 meters below the seafloor (mbsf), where heat from the earth’s mantle sterilizes substrate (D'Hondt et al., 2002; Plümper et al., 2017). This implies a gigantic scale of microorganisms in deep-sea sediments, making it the largest biosphere by volume (Cario et al., 2019). While early approximations of a total sub-seafloor sedimentary biomass of up to 303 gigatons appear to have been overestimated due to extreme variations between different sediment types (Whitman et al., 1998), updated estimates of approximately 4.1 gigatons still indicate a major contribution to global biomass, roughly equaling its seawater and terrestrial counterparts (Kallmeyer et al., 2012).

Despite their low population density, microbial deep marine subsurface communities act on a geological timescale and control the storage of massive amounts of carbon while also exerting influence over global biogeochemical sulfur and nitrogen cycles, among others (Orcutt et al., 2011; D'Hondt et al., 2019; Li et al., 2021). Contrary to early conceptions, the deep marine subsurface is a highly diverse biosphere, with distinct environments having different temperatures, hydrostatic pressures, and electron donor and acceptor availabilities (Schrenk et al., 2010). The microorganisms in those habitats, therefore, require unique adaptations and survival strategies, making them treasure troves of genetic information with biotechnological potential (Jeon et al., 2009; Schröder et al., 2014).

A multitude of studies have investigated sediments that are rich in organic molecules and undergo oxidant depletion with increasing depth (D'Hondt et al., 2004; Biddle et al., 2006; Lever et al., 2010; Inagaki et al., 2015), as well as starved sediments that remain oxic throughout the sediment column down to the basement rock (D'Hondt et al., 2009; Kallmeyer et al., 2012; Røy et al., 2012). In active ocean ridge flanks, fluid circulates beneath the sediment (Fisher et al., 2014), which is heated up geothermally and is chemically altered by interactions with the rock and microbial metabolism occurring in its basement (Boettger et al., 2013; Jungbluth et al., 2013). The exchange of hydrothermal fluid and ocean water is impeded by the above sediment layers, resulting in increased fluid flow within exposed basement rock, where either fresh seawater enters or altered water is expelled (Wheat et al., 2004; Kvenvolden and Rogers, 2005). Between the entrainment and discharge of fluid, diffusion into the overlying sediments can occur. Depending on the amount of alteration the fluid has undergone, various electron acceptors such as oxygen, nitrate, and sulfate are reintroduced into the sediment that would otherwise lack oxidants (Boettger et al., 2013; Orcutt et al., 2013; D’Hondt et al., 2015; Wankel et al., 2015).

**Figure 1:**
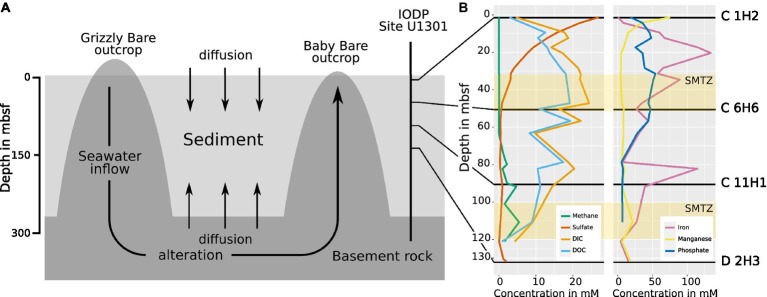
**(A)** Scheme of the hydrogeological regime at the eastern flank of the JdF Ridge. Seawater enters the basement at the Grizzly Bare outcrop and circulates within the basement rock, where it undergoes chemical alteration. Sediment laying above the basement impedes fluid exchange with the ocean, but diffusion into sediment layers still occurs from above and below. At Baby Bare Seamount, the altered seawater from the basement is expelled again. The IODP drill site U 1301 and the depths from which the samples in this study were obtained are indicated (adapted after Engelen et al., 2008). **(B)** The chemical profile of pore water obtained during sampling by IODP in 2004 from cores U1301 C and D. The distribution of sulfate shows diffusion into the sediment both from above and below, while methane is only present when the sulfate concentration in the pore water is minimal, resulting in the formation of two sulfate methane transition zones (SMTZs) at ~30–50 m and at ~100–120 m (Fisher et al., 2005a) indicated as yellow bars. DIC: dissolved inorganic carbon. DOC: dissolved organic carbon. Data made available by IODP in 2008 (Ocean Drilling Data, 2021).

The effects of oxidant recharge from basement rock into deep-sea sediments on microbial communities are currently not well understood (Biddle et al., 2005; Huber et al., 2006; Labonté et al., 2017; Zinke et al., 2018), even though they are likely a common feature of active ocean ridge flanks with significant influence on global biogeochemistry (Davis E. E. et al., 1996). One of the best-studied active ridge systems is the Juan de Fuca (JdF) ridge and its flanks, which have been the destination of several expeditions dedicated to the study of its hydrogeology (Davis E. et al., 1996; Fisher et al., 2004; Fisher, 2010). The JdF ridge features fluid circulation driven by geothermal heat within its basement, with discharge and replenishment through several basement outcroppings (Wheat and Mottl, 2000; Hutnak et al., 2006; Figure 1A). At the same time, altered ocean water diffuses from the crust into the overlaying sediment layers and replenishes oxidants (Huber et al., 2006). The interaction of microorganisms in the sediment, hydrothermal fluid diffusing from below, and ocean water above causes sulfate depletion and methane accumulation in the sediment at depths between ~30–50 mbsf and ~100–120 mbsf, which are so-called sulfate methane transition zones (SMTZs; Figure 1B; Fisher et al., 2005a). The microbes present within the sediment layers and their influence on the local geochemistry have become an important subject of research in recent years (Biddle et al., 2005; Huber et al., 2006; Engelen et al., 2008; Reese et al., 2018; Zinke et al., 2018), and model systems such as the JdF ridge show that depth and sediment type influence microbial composition, even though no microbes from the fluid migrate to the basement (Labonté et al., 2017). However, since, so far, only cultivation-dependent techniques (Biddle et al., 2005) and 16S rRNA amplicon sequencing (Labonté et al., 2017) have been used to study these communities, there is still only limited information on metabolic genes and, therefore, on the metabolism of the microbes living in this habitat.

Recently, fluorescent activated cell sorting (FACS) and next-generation sequencing have been used to unravel the genomes of microorganisms occurring in rock and sediment of the Atlantic ridge, where fluid circulation also occurs (Goordial et al., 2021). However, this strategy might offer only limited views into the microbial community structure and metabolism because it misses extracellular DNA and introduces bias through multiple displacement amplification, which is required for sequencing the low amounts of DNA obtained from the sorted cells (Clingenpeel et al., 2014; Direito et al., 2014; Sabina and Leamon, 2015; Stepanauskas et al., 2017; Kaster and Sobol, 2020; Sobol and Kaster, 2023). Metagenomics without prior DNA amplification, on the other hand, has not yet been successfully employed on sediment samples from deeper than 50 mbsf. Major reasons for this include limited sample availability, extremely low cell counts (Kallmeyer et al., 2012), and the difficult sediment lithology of sub-seafloor sediments, which limit DNA extraction yields (Hurt et al., 2001) or result in the co-extraction of detrimental substances that could inhibit downstream sequencing processes (Lever et al., 2015).

In this study, we optimized DNA extraction protocols to successfully perform shotgun metagenomics on bulk-extracted DNA from four selected sub-seafloor sediment samples obtained close to the Baby Bare seamount within the JdF ridge during the 2004 Integrated Ocean Drilling Program (IODP) expedition 301 (Supplementary Image 1). We present, to the best of our knowledge, the first non-amplified and, therefore, unbiased shotgun metagenomic datasets obtained from sub-seafloor sediment samples following a vertical transect through the sediment from 1.5 to 132.2 mbsf, where basement fluid reintroduces sulfate. This study gives an overview of the community composition and changes above, within, between, and below the SMTZs, as well as the metabolic potential of the microbes living in these sediment layers. Furthermore, the reconstructed metagenome-assembled genomes (MAGs) show strong evidence for the presence of Asgard archaea, which have previously not been reported in JdF sediments, as well as many currently unknown taxa. The data were also used to infer the stratification of microbial metabolic functions in the deep-sea sediment of a site with electron acceptor recharge via basement fluid at the JdF ridge for the first time.

## Methods

### Site description, sample description, and DNA extraction

The deep-sea sediment samples used in this study were taken during IODP Expedition 301 in August 2004 on the eastern flank of the Juan de Fuca (JdF) ridge, approximately 2667.4 meters below the sea surface (Fisher et al., 2005b). A description of the samples is given in Table 1, and details of the chemical profiles are provided in Figure 1. Horizontal sections were cut from sediment cores obtained from holes “C” (47° 45′ 16.8″ N; 127° 45′ 48.0″ W) and “D” (47° 45′ 16.6″ N; 127° 45′ 46.8″ W) of site 1,301 (Supplementary Image 1), which are located within 20 meters from each other and capture the whole sediment column from the seafloor to the basement (Fisher et al., 2004). Both holes were cored using an advanced piston corer. The operations of coring the holes are detailed in the site report published by the IODP (Fisher et al., 2005a). After drilling, the cores were taken from the core catcher and placed on a horizontal rack, cut by wire, and transferred onto shipboard cold storage (Fisher et al., 2005b). All samples were kept at −80°C until DNA extraction was performed.

**Table 1:** Properties of the samples used for this study and results of DNA extraction with the improved protocol outlined in the Supplementary information.

Sample	C 1H2	C 6H6	C 11H1	D 2H3
Hole	C	C	C	D
Depth (mbsf)	1.5	50.6	90.6	132.2
Methane (mM)	0.00	0.00	4.70	NA
Sulfate (mM)	27.00	16.23	0.95	2.02
DOC (mM)	2.84	10.88	11.19	0.63
Remarks	Topmost sample	Upper SMTZ	Anaerobic layer	Basement influenced
DNA (ng/μl)	3.820	0.080	0.100	0.453
Total extracted DNA (ng)	95.5	1.6	2.0	11.3
extracted DNA/sediment (ng/g)	22.74	0.32	0.40	2.11

The presented information on depth and pore water composition was published at the core data repository of the IODP in 2008 (Ocean Drilling Data, 2021). The DNA concentration of the eluate was determined using the Qubit™ 4 Fluorometer from Invitrogen™. mbsf, meters below seafloor; DOC, dissolved organic carbon; NA, data not available.

For the DNA extraction, approximately 5 g of sediment was available for each sample. Before DNA extraction, each sediment sample was pre-washed using a protocol (Fang et al., 2015). The DNA extraction protocol used a FastDNA™ SPIN Kit for Soil (MP Biomedicals, California, USA), which was adapted from Kaster et al. with some alterations (Kaster et al., 2014; Supplementary Data Sheet 1). DNA concentrations were determined using a Qubit TM 4 fluorometer (Invitrogen, Massachusetts, USA) and its high-sensitivity kit.

### Sequencing, assembly, and binning of MAGs

Libraries were created using the NEBNext® Ultra™ II FS DNA Library Prep Kit for Illumina® Sequencers (Illumina, California, USA) following the manufacturer’s protocol for inputs below 100 ng DNA. For C 1H2 and C 11H1, a DNA input amount of 1 ng was used in combination with 12 PCR cycles during the barcoding step of the library preparation. Due to residual inhibitory substances in C 6H6 and D 1H2, input DNA amounts had to be reduced to 0.1 ng, and PCR cycles were increased to 14 for these two samples. Sequencing was performed using the Illumina® NextSeq550 platform (Illumina, California, USA) with a paired-end high-output v2.5 chemistry kit (2 × 150 cycles). Sequencing read quality was assessed using FastQC (Andrews, 2010). Quality-filtering and adapter trimming were performed 2-fold using Trimmomatic (Bolger et al., 2014) with the following settings: “SLIDINGWINDOW:4:15 LEADING:5 TRAILING:5 ILLUMINACLIP:2:30:10 MINLEN:70” and then BBDuk (Bushnell, 2020) with “minlength = 70 mink = 11 ktrim = r.” Overlapping read pairs were then merged using FLASH (Magoč and Salzberg, 2011) with settings: “-m 10 -M 150-X 0.02.” The processed sequence read data of all metagenomic samples were co-assembled using Megahit (Li et al., 2015) with a k-list ranging from 21 to 141 bp in increments of 10 bp. Coverage profiles of the metagenomes were created by short-read mapping using BamM (BamM: Working with the BAM, 2016) and SAMtools (Li et al., 2009). Approximately 0.5% of all contigs were removed by subtracting BLAST hits against the human genome GRCh38 with more than 95% identity and 70% query coverage. A summary of the sequencing data can be found in Supplementary Table 1. All data were deposited in the National Centre for Biotechnology Information (NCBI) Sequence Read Archive (SRA) under the accession number PRJNA901380.

MAGs were binned from the metagenomes using MaxBin2 (Wu et al., 2016), CONCOCT (Alneberg et al., 2013), and Metabat2 (Kang et al., 2015), and subsequently de-replicated and de-aggregated using DAS-Tool (Sieber et al., 2018). MAGs were then filtered for completeness and contamination with CheckM (Parks et al., 2015) and screened for misattributed/contaminating contigs using MDMcleaner, which were then removed (Vollmers et al., 2022). Only MAGs with completeness scores >50% and contamination estimates <5% were selected for further analyses. Phylogenetic relationships between MAGs were determined using GTDB-TK (Chaumeil et al., 2019).

### Taxonomic and metabolic analyses

16S and 23S rRNA sequences were identified in the assembled contigs using the get_markers function of MDMcleaner (Vollmers et al., 2022) and were classified using the QIIME2 pipeline (Bolyen et al., 2019) by training a classifier with the “fit-classifier-naïve-bayes” parameter on the V3/V4 16S rRNA gene sequences of SILVA database version 132 (Quast et al., 2013). It was then used to classify the predicted rRNA genes with the classify-sklearn parameter. Diversity analysis was conducted with the Python package ExpressBetaDivertity (Parks and Beiko, 2012) on predicted 16S rRNA sequences, which were treated as operational taxonomic units (OTUs) for diversity analysis. To display alpha diversity, the Shannon index was calculated to weigh high-abundance taxa higher, while the ACE index was calculated to weigh low-abundance taxa higher. Beta diversity was calculated with the Jaccard index to compare the abundances of specific OTUs between samples. Furthermore, the predicted OTUs were used to calculate the Good’s coverage to estimate the completeness of the datasets. Classified 16S rRNA genes were combined with the mapping data of every sample. Potential protein-coding sequences were identified using Prodigal (Hyatt et al., 2010), and conserved single-copy marker genes were extracted with FetchMG (Sunagawa et al., 2013). All protein sequences were aligned with NCBI GenBank Release 235 using Diamond (Buchfink et al., 2015). Alignment information and coverage profiles of all discovered and classified genes were combined in order to create visual representations of the microbial diversity of each sample using KronaTools (Ondov et al., 2011).

Functional classification of protein-encoding genes was performed using the entries from the database of the eggNOG mapper called Orthologous Groups (OGs; Huerta-Cepas et al., 2019) and combined with the mapping data of each metagenome sample using R Studio (Team R, 2020) and visualized with the ggplot2 package (Wickham, 2016) of R. Genes relating to selected metabolic processes in the sediment were then filtered according to a list of 206 key marker genes and their corresponding “KEGG Orthology” (KO) identifiers, which were compiled from previous studies (Supplementary Table 2; Lever et al., 2010; Galperin et al., 2012; Barco et al., 2015; Acinas et al., 2021; Bell et al., 2022). The completeness of metabolic pathways was determined using the OGs listed in Supplementary Table 2. To calculate pathway completeness, hits for OGs predicted by the eggNOG mapper (Huerta-Cepas et al., 2019) were checked to see whether they could be found on a MAG contig or not. Hits found in MAGs were then compared to the pathway modules from Supplementary Table 3. In cases where several combinations of OGs could constitute a pathway, the highest pathway completeness was considered.

## Results

### Taxonomic and diversity analyses of prokaryotic communities in JdF deep-sea sediments

The modified FastDNA extraction protocol succeeded in obtaining DNA yields ranging from 0.30 to 22.74 ng DNA per gram of sediment, for a total of 1.6 to 95.5 ng DNA (Table 1). The resulting extraction rates do not correlate with the cell counts reported for the corresponding sampling depths (Fisher et al., 2005a). This discrepancy is largest for the topmost sample C 1H2, which had the highest DNA extraction rate but the lowest reported cell count (1.4 × 10^8^ cells/cm^3^ at 1.5 mbsf), and the anaerobic sample C 11H1, which had the lowest DNA extraction rate but the highest reported cell count (5.8 × 10^8^ cells/cm^3^ at 91.7 mbsf).

**Figure 2:**
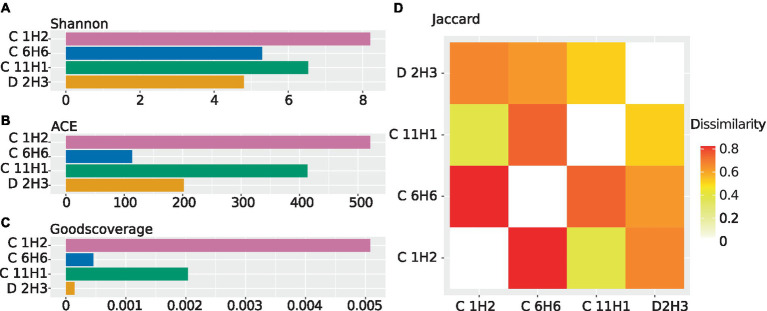
Alpha and beta diversities calculated from the abundance of contigs carrying 16S rRNA genes using ExpressBetaDiversity (Parks and Beiko, 2012). The 16S rRNA sequences used for diversity analysis were predicted from the contigs of the co-assembled metagenomes, and abundances were estimated by mapping the individual sample’s reads back onto the co-assembly. **(A)** Shannon diversity as a barplot. The diversity within the samples is shown while considering species abundance. The Shannon index weighs the abundance of highly abundant species higher. **(B)** ACE diversity as a barplot. The diversity within the samples is shown while considering species abundance. The ACE index places an additional focus on less abundant species. **(C)** Good’s coverage as a barplot. The estimated completeness of a sample is displayed as the negative decadic logarithm of the Good’s coverage, meaning that lower values indicate higher estimated completeness. **(D)** Jaccard diversity for every sample to every other sample as a heatmap. Displayed is the inverse of the similarity between every sample, meaning that two identical samples would have a dissimilarity of “0”.

Sequencing resulted in four metagenomes (Supplementary Table 1). Co-assembly yielded 2,586,028 contigs in total. The alpha diversity of the topmost sample C 1H2 (1.5 mbsf) was the highest, followed by the anaerobic sample C 11H1 (90.6 mbsf). According to the Shannon metric, the diversity of the basement-influenced sample D 2H3 (132.2 msbf) is the lowest (Figure 2A), while the ACE metric ranks sample C 6H6 in the first SMTZ (50.6 mbsf) as the least diverse (Figure 2B). The Good’s coverage shows that the discovered OTUs cover the diversity within the sediment almost entirely. C 1H2 is the least complete sample; however, it still has a high coverage of 99% (Figure 2C). Using the Jaccard index, which disregards abundances, C 1H2 and C 6H6 (Figure 2D) are the least similar.

The 16S rRNA gene prediction using MDMcleaner (Vollmers et al., 2022) yielded 567 sequences. All were classified with confidence of approximately 70% by the QIIME2 (Bolyen et al., 2019) pipeline based on a naïve Bayesian model trained on V3/V4 16S rRNA regions from the SILVA database release 132 (Quast et al., 2013). The classification of predicted 16S rRNA genes shows that 54% of bacterial rRNAs from C 1H2 (1.5 mbsf) and 45% from C 6H6 (50.6 mbsf) could not be placed into a known phylum.

**Figure 3:**
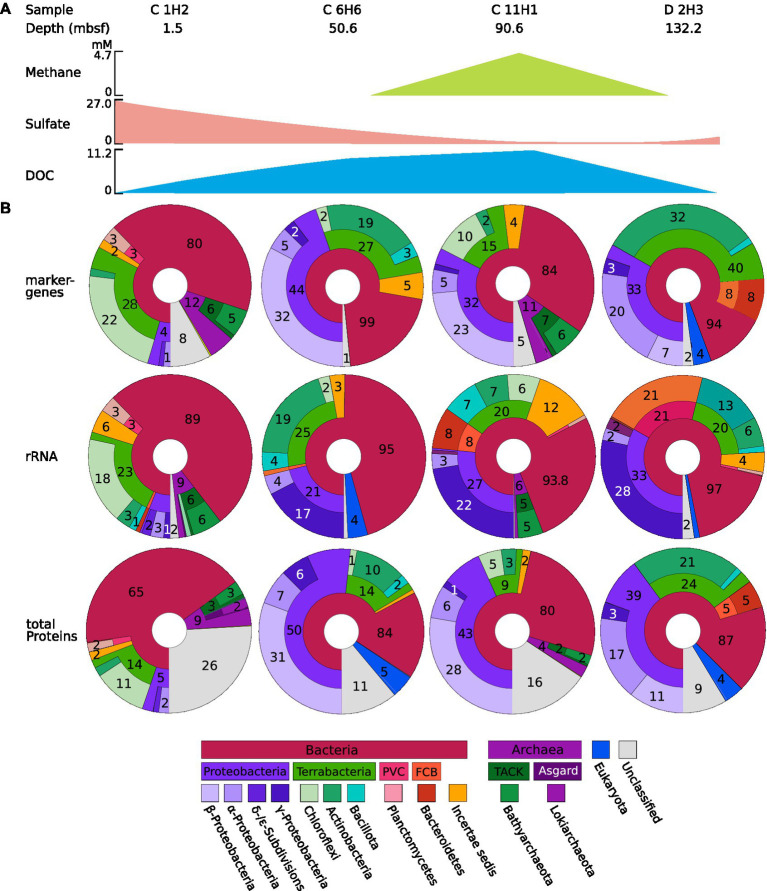
**(A)** Information on the sample and key chemical components. The concentrations of methane, sulfate, and dissolved organic carbon (DOC) are shown as a simplified scheme; for a more detailed graph, see Figure 1. **(B)** Microbial abundance profiles of the four samples C1H2, C6H6, C11H1, and D2H3 based on rRNA, protein, and marker gene classification from IODP site U1301. Marker genes, 16S rRNA, and protein predictions were performed using the co-assembled metagenome, and their abundance within the original sample was determined using short-read mapping data. Classifications were obtained using the QIIME2 (Bolyen et al., 2019) classification outlined in the methods for 16S rRNA and by alignment against NCBI GenBank Release 235 for total protein predictions and marker gene predictions. Displayed are the classifications of identified sequences up to domain, phylum, and class, sequences that cannot be classified are displayed as part of the next higher level with no label. Sequences that cannot be attributed to a Domain are labeled as “unclassified.” CPR = Candidate Phyla Radiation. The Krona charts corresponding to the depicted figures can be found in Supplementary Data 2–4.

The community composition of the observed taxa shifts drastically between the samples, as indicated by the beta diversity (Figure 2D). Based on 16S rRNA gene analyses, the most abundant known phylum in C 1H2 (1.5 mbsf) is Chloroflexota (18% of 16S rRNA genes), represented almost entirely by the two classes Dehalococcoidia (14% of 16S rRNA genes) and Anaerolineae (3% of 16S rRNA genes). In the deeper samples C 6H6 (upper SMTZ, 50.6 mbsf), C 11H1 (anaerobic layer, 90.6 mbsf), and D 2H3 (basement-influenced, 132.2 mbsf) Proteobacteria become dominant, with a 21, 27, and 33% relative abundance, respectively. Archaeal SSU rRNA genes were only found in samples C 1H2 and C11H1, wherein the most abundant identified archaeal phylum is Bathyarchaeota (6 and 5% of total abundance, respectively). Most archaeal signatures classified with QIIME2 (Bolyen et al., 2019) were similar to sequences from marine environmental samples, but the majority could not be placed within a known class. Other more abundant phyla are Actinobacteria, Bacteroididetes, Bacillota, Planctomycetes, and the members of the Candidate Phyla Radiation (CPR) Atribacteria, Patescibacteria, and Aerophobetes (Figure 3; Supplementary Data Sheet 2–4). Tables of the marker gene annotations, 16S rRNA annotations, and protein annotations are shown in Supplementary Data Sheet 2–4.

### Predicted protein encoding genes show the stratification of metabolic potential through the sediment

From the co-assembly of all sediment samples, 3,563,725 potential protein-encoding genes were predicted using Prodigal (Hyatt et al., 2010). Almost half of these (1,711,831; 48%) could be assigned to orthologous groups (OGs) of the EggNOG database using the Hidden Markov Model (HMM)-based eggNOG mapper (Huerta-Cepas et al., 2019) and 2,562,388 (72%) aligned with the NCBI env_nr with an E-value below 0.01. In contrast, only 336,776 (9.5%) of the predicted protein-encoding genes could be aligned against sequences in the NCBI-nr database (E-value cutoff 0.01).

**Figure 4:**
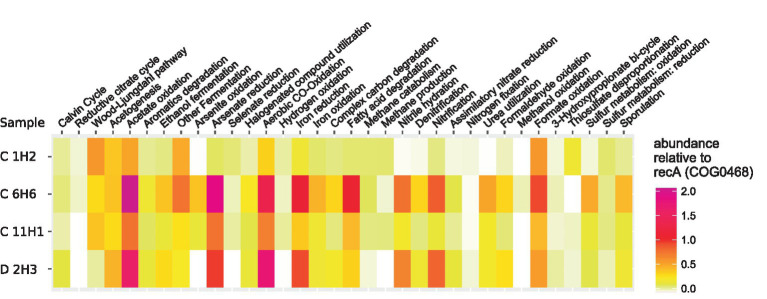
Heatmap of KO-Numbers collated into relevant functions associated with detected orthologues. OGs were predicted from the co-assembled metagenomes with the eggNOG Mapper (Huerta-Cepas et al., 2019) and their abundance was calculated by mapping the information of the corresponding encoding contigs back to the reads. Displayed is the relative abundance of the orthologues in each sample, normalized to the abundance of the universal housekeeping gene/function COG0468 (recA). In cases where closely related OGs were grouped into more general metabolic functions, the corresponding mean value is shown. Functional groups with a total relative abundance below 0.02 have been removed for better representability.

Overall, for 190 of the 209 selected OGs from the KEGG orthology (KO) database (Supplementary Table 2), hits were detected in the metagenome assemblies, which represent key genes of selected pathways. If feasible and sensible, the detected pathways were grouped into metabolic and other functions relevant to deep-sea sediments and are presented in Figure 4. Genes for acetogenesis as well as the Wood–Ljungdahl pathway were highly abundant throughout the sediment column. OGs for methane production and methane catabolism were found at a higher abundance in C 1H2 and C 11H1 (90.6 mbsf), while only OGs for methane catabolism were more abundant in C 6H6. OGs for sulfur compound reduction were less abundant in the deeper samples than in the topmost sample, C 1H2 (1.5 mbsf). In sample C 6H6 (50.6 mbsf) and the deepest sample D 2H3 (132.2 mbsf), OGs for the oxidation of sulfur and iron were more abundant than in the samples above. The highest relative abundance of OGs for nitrification and denitrification was also found within these deep samples.

### Assembly and binning of sequencing data into MAGs

Assembly and de-aggregation resulted in 209 bins belonging to 18 different phyla. From those bins, 42 were selected as MAGs for further analyses based on quality criteria of at least 50% completeness and contamination estimates of 5% or less. Of the 62 investigated pathways, 60 were represented in at least one MAG in the form of a corresponding EggNOG OG. Detailed information on all MAGs is given in Supplementary Table 4. The pathway completeness of every MAG is given in Supplementary Table 5.

A total of 11 MAGs were assigned to the phylum Chloroflexota, of which 2 belong to the class Anaerolineae and 9 to the class Dehalococcoidia. Genes associated with the Wood–Ljungdahl pathway occurred more frequently (4 of 11 MAGs) in the discovered Chloroflexota MAGs than in the other MAGs (6 of 31 MAGs). All Chloroflexota MAGs shared a high pathway completeness for methanogenesis from acetate. Orthologues for methanogenesis from acetate were also found in the Bathyarchaeon MAG (JdF_42) with 100% module completeness (Supplementary Table 5).

**Figure 5:**
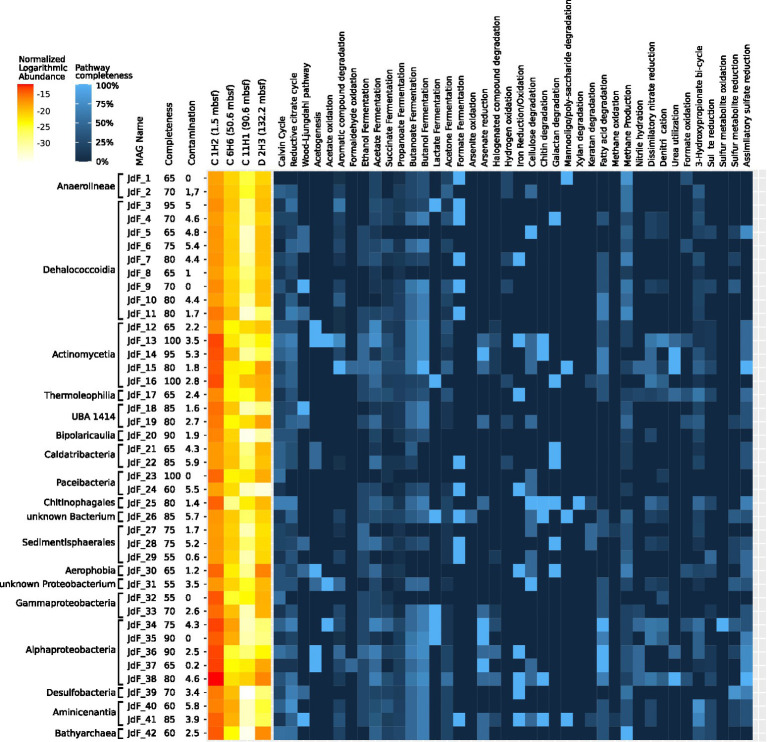
Metabolic profile of MAGs. The relative abundance of each MAG was determined based on short-read mapping information and is displayed as a heat map on the left side of the plot. The heat map shows the pathway completeness within the 42 selected metagenome-assembled genomes (MAGs) obtained from Juan de Fuca (JdF) samples that have more than 50% estimated completeness and less than 5% estimated contamination. To predict pathway completeness, OGs were predicted from the co-assembled metagenome using eggNOG Mapper (Huerta-Cepas et al., 2019) and checked to see whether they belonged to a MAG contig. OGs of MAGs were compared to the pathway modules outlined in Supplementary Table 3, and the number of orthologues found in a MAG is displayed as a percentage. Taxonomic classifications are based on GTDB-TK (Chaumeil et al., 2019) and the order in which MAGs are listed reflects the respective maximum likelihood-based phylogenetic relationships of the selected MAGs. Completeness and contamination estimates were obtained using MDMcleaner (Vollmers et al., 2022).

Of the eight Proteobacteria MAGs, five belonged to Alphaproteobacteria and three belonged to Gammaproteobacteria. When compared to C 1H2, Alphaproteobacteria of the order Rhizobiales showed an increased abundance in D 2H3. These microorganisms have been described in the JdF Ridge Flank sediments before (DeLong, 2005; Reese et al., 2018), and their distribution as well as the analysis of MAGs suggests that they may engage in acetate oxidation and acetogenesis (Figure 5).

A stratification for sulfate-metabolizing microorganisms along the chemical stratification outlined in the introduction was observed. According to 16S rRNA gene analysis, *Desulfobacteria* were found in the topmost sample C 1H2 at 2% of total 16S rRNA gene abundance and in the basement-influenced sample D 2H3 at 1% of total 16S rRNA gene abundance. Their abundance dropped within the anaerobic layer sample C 11H1 to 0.7% and could not be detected within the SMTZ sample C 6H6. MAG JdF_39, which was taxonomically most closely related to a Desulfobacterium, had a pathway completeness of 80% for dissimilatory sulfate reduction.

The taxonomic classification of MAG JdF_26 was unclear since MDMcleaner (Vollmers et al., 2022) results indicated members of the Chloroflexota as the most closely related reference genomes, while GTDB-TK and 16S rRNA-based SINA assessments suggested an association with the Candidate Phylum Ca. Mcinerneyibacteriota. JdF_26 is, however, interesting since it was the only MAG with OGs for the arsenite oxidase subunit *aoxB* (ko:08356). Furthermore, it harbored OGs for formate and lactate fermentation and several OGs for complex carbon degradation, including chitin degradation (Figure 5), suggesting that it might obtain energy from the breakdown of complex carbon and the fermentation of the obtained sugars. The other enigmatic MAG (JdF_31) is likely a Proteobacterium but could not be assigned any further by GTDB-TK (Chaumeil et al., 2019), although MDMcleaner (Vollmers et al., 2022) suggested its contigs to be most closely related to the Gammaproteobacterium *Paraburkholderium ferrariae*, however, with a very low fraction of trusted base pairs. It had OGs for sulfate reduction (*aprA*; ko: K00394 and *sat*; ko:K00958), cellulase (ko:K01779), acetate symporter *actP* (ko:K14393), and the acetogenesis pathway OG *ackA* (ko:K00925; Figure 5), suggesting that it might be a sulfate reducer capable of using sugars from cellulose and acetate as electron donors.

The PVC phylum (Wiegand et al., 2020) was mainly represented by the planctomycetal classes Pirelluales and Phycisphaeraceae in the topmost sample C 1H2 (1.5 mbsf) and Pirellulales in the deepest, basement-influenced sample D 2H3 at 131.78 mbsf. Pirelullales members have been found to possess the necessary genes for C1 compound degradation as well as respiring metabolism (Glöckner et al., 2003), and corresponding MAGs were only found in the topmost and basement-influenced JdF samples, but not in any layers between. The Pirellulales of the basement-influenced layer D 2H3 are possibly descendants of ancestors who were buried closer to the active ridge, which would make them evolutionarily distinct from, but still directly related to those of the topmost layer C 1H2 (Starnawski et al., 2017). Other bacterial MAGs found in all samples belong to the PVC superphylum and the so-called Candidate Phyla Radiation (CPR), commonly used to describe taxa with no cultured representatives (with a few exceptions; Castelle et al., 2018; Wiegand et al., 2021). The most complete bacterial MAG reconstructed from the JdF metagenomes, with a total amount of 677,305 bp and a completeness of 100% according to MDMcleaner, JdF_23 was found to belong to the Candidate Phylum Paceibacteria, for which a reduced metabolic potential and genome size have been described before (Singleton et al., 2021). To sustain their lifestyle, the discovered Paceibacterium might rely on syntrophy or parasitism to sustain its lifestyle (Chiriac et al., 2022). Three MAGs, which were most closely related to the order *Sedimentisphaerales* according to GTDB-TK (Chaumeil et al., 2019), might live as detrivores within the shallow sediment since they have OGs for cellulose degradation such as beta-glucosidase (ko:K05350) and endoglucanase (ko: K01179). One MAG of a bacterium belonging to the CPR and potentially living as a detrivore in the upper sediment layers was closest related to *Sediminibacterium magnilacihabitans* (JdF_25), which had many gene OGs for complex carbon degradation and OGs for nitrate and nitrite reductases. It was one of the highest quality MAGs, with an estimated completeness of 80% and contamination of 1.4% according to CheckM (Parks et al., 2015) as well as a bin trust of above 0.95 according to MDMcleaner (Vollmers et al., 2022). Classification with GTDB-TK (Chaumeil et al., 2019) placed it closest to the order Chitinophagales, in particular to *Sediminibacterium magnilacihabitans*. JdF_25 had the biggest array of OGs for complex carbon degradation, including chitinase (ko:K01183) and the only found instance of Xylan 1,4-beta-xylosidase (*xynB*; ko: K01198) as well as multiple OGs for cellulose degradation. It also had OGs for nitrate reductase (*nirB*; ko:K00362, *nirD*; ko:K00363) and denitrification (*norC*; ko:K02305, *nosZ*; ko:K00376; Figure 5). It is likely that it uses complex carbohydrates such as cellulose and chitin as electron donors and nitrate or nitrite as electron sinks. The MAG JdF_30 is also a member of the CPR and closest related to the class Aerophobales, according to GTDB-TK (Chaumeil et al., 2019). It had a completeness of 65% and a contamination of 1.2%, according to CheckM (Parks et al., 2015). With its relatively low contamination, it is an interesting candidate to investigate the metabolic potential of this understudied phylum. It had several OGs necessary for acetogenesis (*ackA*; ko:K00925, *pta*; ko:K00625), acetate fermentation, an OG for iron oxidation (*bfr*; ko:K03594), and the Wood–Ljungdahl pathway (*cooS*; ko:K00198; Figure 5). It also had several OGs, which might enable iron-coupled anaerobic oxidation of methane, which is one of the major reactions of oxidizing methane within sediments (Yang et al., 2021).

## Discussion

### Improved DNA extraction enables shotgun metagenomics of deep marine oligotrophic sediment

The deep sea hosts a very diverse and difficult-to-access biosphere, which we are only beginning to understand (Sogin et al., 2006; Cario et al., 2019; D'Hondt et al., 2019). Metagenomics has become an indispensable tool for studying the composition and metabolic potential of microbial life in general and also in deep-sea sediments. However, low cell counts, inhibiting substances such as humic acids, and sample scarcity pose challenges for metagenomics studies of this often oligotrophic environment (Zipper et al., 2003; Kallmeyer et al., 2012). Hence, no previous shotgun metagenomes for sediment samples deeper than 50 mbsf have been reported that did not involve a DNA amplification step prior to library preparation. DNA amplification methods such as multiple displacement amplification (MDA) can be used to obtain metagenomes from sediment that would otherwise be inaccessible to metagenome sequencing due to low cell counts and, therefore, low DNA extraction yields (Biddle et al., 2011). Using MDA to increase the amount of DNA can, however, introduce bias (Sobol and Kaster, 2023). Single-cell genomics can be used as a complementary method to counter low DNA availability, but it also has its challenges (Yilmaz et al., 2010; Kennedy et al., 2014; Sabina and Leamon, 2015; Liu et al., 2020).

The first shotgun metagenomics data obtained from bulk-extracted DNA of deep marine sediment below 50 mbsf and even 100 mbsf were achieved using an optimized DNA extraction protocol, which succeeded in recovering more than 1 ng of DNA from all samples using approximately 5 grams of sediment. Due to the low amount of sampling material available, the obtained DNA was only sufficient to generate the presented metagenomes. No single-cell amplified genomes could be generated since all samples were used for DNA extraction and had not been stored in glycerol to preserve the cells. Considering that it is now feasible to prepare libraries using DNA inputs as low as 10 pg (Hirai et al., 2017), this study might enable shotgun metagenomics from even more oligotrophic sites, such as habitats below oceanic gyres, especially when more than 5 g of sampling material is available. In conjunction with single-cell and amplicon sequencing, our method could enable more unbiased analyses of currently understudied deep-sea sediments and reveal novel organisms and metabolic pathways that have previously been missed.

The observed DNA extraction rates do not correlate with the cell counts based on acridine orange staining reported by Fisher et al. (2005a). The method of acridine orange staining likely results in overestimated cell counts due to residual DNA persisting after cells have perished (relic DNA; Luna et al., 2002). This should lead to increased DNA extraction rates, yet the highest DNA extraction rate was observed when the lowest cell count was reported. One possible explanation could be that less relic DNA was extracted from the samples with higher reported cell counts. Humic acids, which are common in marine sediment (Fooken and Liebezeit, 2000), have a strong absorptive effect on DNA (Saeki et al., 2011). The protocol presented here uses a prewashing step to remove inhibiting substances before DNA extraction, which might remove humic acids and relic DNA alike, leaving only the DNA from intact cells in the sample for extraction. Furthermore, the FastDNA extraction kit might positively select DNA from intact cells over relic DNA bound to humic acids. These factors could result in a reduced DNA extraction rate when the ratio of living to dead cells in a sample is low and humic acids are present, which would explain the observed discrepancy.

A total of 54% of all 16S rRNA gene sequences in sample C 1H2 were assigned to the domain Bacteria but could not be further assigned to any known bacterial phylum, potentially representing entirely new lineages (Figure 3B) and thereby also reflecting the lack of reference data for these undersampled habitats. Most of the abundance discovered in the topmost sample belongs to Chloroflexota. This is in accordance with previous studies, which have frequently reported them to be dominant in deep-sea sediments (Inagaki et al., 2006; Durbin and Teske, 2011; Kaster et al., 2014; Jungbluth et al., 2016; Sewell et al., 2017). This is likely due to their ability to degrade complex carbohydrates while reducing CO_2_ to acetate, allowing them to utilize a diverse pool of complex carbon molecules and perform anaerobic acetogenesis in shallow sediments (Fincker et al., 2020), i.e., using the Wood–Ljungdahl pathway, which enables the use of electron donors even in more oligotrophic sediments (Liu et al., 2022). Previous 16S rRNA amplicon studies of JdF flank sediment from the nearby site U1362, which is located at the Grizzly Bare outcrop (Supplementary Image 1), showed a much higher abundance of Chloroflexota [60.3–86.7% compared to 1–18% reported here (Figure 3; Labonté et al., 2017)]. This discrepancy is likely due to PCR bias that can be introduced in amplicon studies depending on PCR conditions and primer specificity and often leads to potential over-or underestimation of different taxa (Kennedy et al., 2014). Potential novel 16S rRNA lineages, which were uncovered by our shotgun metagenomics approach, are likely not covered by standard 16S rRNA amplicon primers.

In addition to many novel rRNA sequences, the MAGs JdF_26 and JdF_31 likely represent the first genomes of novel taxa with relatively high-quality scores, according to MDMcleaner (Supplementary Table 4). Jdf_26 is the only MAG in the presented data with an arsenite oxidase, and Jdf_31 possesses a cellulase OG. Since deep-sea microbes are adapted to high pressures and low substrate concentrations within the sediment, these enzymes could be interesting targets for bioprospecting with potential applications in bioremediation and bioethanol production (Jeon et al., 2009; Schröder et al., 2014; Jayasekara and Ratnayake, 2019; Kabiraj et al., 2022). Overall, only 9.5% of the predicted protein-encoding sequences could be aligned to characterized protein-encoding genes with an e-value below 0.01, indicating that although some functional information could be assigned based on structural information contained in HMMs, the majority of genes nonetheless represent novel variants with low sequence similarity to any previously reported genes. This shows an enormous knowledge gap regarding so-called hypothetical proteins, also referred to as functional dark matter (Ardern et al., 2023). For up to 26% of these, the query yielded multiple hits to Archaea and Bacteria in the nr database, making taxonomic classification ambiguous. They are, therefore, marked as “unclassified” (Figure 3C).

The majority of Chloroflexota MAGs have a high module completeness for methanogenesis from acetate. This is, however, not necessarily an indication of methanogenic potential in the newly discovered Chloroflexota MAGs: Bathyarchaeota, which represent the majority of classified archaeal rRNA gene sequences in C 11H1 and C 1H2, are known to share the ability to reduce CO_2_ with members of Anaerolineae and Dehalococcoidia within the observed sediment, although via a different mechanism/pathway (He et al., 2016). They likely conduct acetogenesis through a non-canonical coupling of the acetogenic pathway to the methyl branch of the Wood–Ljungdahl pathway (Farag et al., 2020) and methanogenesis (Evans et al., 2015). The corresponding EggNOG OGs identified in the Chloroflexota MAGs may, therefore, just represent CO_2_ reduction pathways.

Apart from the aforementioned Bathyarchaeota, Asgard archaea, whose occurrence in JdF_ridge flank sediments has not yet been discussed, have been found under the name “Marine Benthic Group B” before (Jungbluth, 2014), but only via 16S rRNA gene amplicon sequencing. This study is, therefore, the first to publish genomic insights into Asgard Archaea from this location. Previous studies have shown Archaea to be an important but often overlooked domain of the deep-sea community (Biddle et al., 2008). Almost all Asgard archaeal sequences presented here belong to the classes Lokiarchaea and Heimdallarchaea and are exclusively located in the topmost layer C 1H2 (Figure 3). They were previously reported to engage in the complete mineralization of aromatic compounds by means of syntropy with nitrite-, sulfite-, and nitrate-respiring microbes (Farag et al., 2020). This dependency on syntropic respiring partners might explain why Asgard archaea are absent in the anaerobic sample C 11H1 at 91.7 mbsf since their syntropic partners would lack the necessary oxidants to support the Archaea. In the deepest sample, D 2H3 at 131.78 mbsf oxidants are available again, but the sediment is highly oligotrophic, so the complex carbohydrates needed by the Asgard archaea might be too depleted for them to remain viable.

Functional assignments of protein-coding genes also help to shed light on the microbial communities and their metabolic potential within the entire sediment column at the JdF Ridge. In the topmost layer (C 1H2), electron acceptors are already depleted to the point where sulfate has become the most viable terminal acceptor, with electron donors in the form of organic carbon compounds still available (Fisher et al., 2005a). In even deeper, older sediment, sulfate depletes gradually under continued microbial activity, creating an environment of changing redox state for the microbes within the upper SMTZ sediment (sample C 6H6 at 50.4 mbsf) followed by anaerobic sediment below (sample C 11H1 at 91.7 mbsf). The active ridge flank below the sampling site causes an upwelling of oxidant-rich water into sediment near the basement interface, preventing its depletion in the deepest sediment layers (sample D 2H3 at 111.2 mbsf) below the secondary SMTZ, which has an inverted pattern compared to the upper SMTZ. In this basement-influenced, deep sediment, carbon molecules are depleted, resulting in conditions similar to those found in sediment beneath oceanic gyres such as the South Pacific Gyre (D'Hondt et al., 2009), with the notable exception that sulfate serves as the final oxidant rather than oxygen. The presented data show that this chemical stratification coincides with the stratification of biological functions and with microbial community shifts. The abundance of OGs associated with sulfate reduction is highest in the strata with the highest reported sulfate concentration, and sulfur compound oxidation OGs are lowest within the anaerobic stratum sample. In the basement, fluid-influenced sample OGs associated with sulfate reduction occur again at an increased percentage, showing that the abundance of these OGs follows the chemical gradient of sulfate. The taxonomic analysis shows that in addition to functional genes, microbial composition changes with this profile not only on a community level but also on the level of individual species, indicating that the functional potential might be tied to taxonomy. Considering that microbes influence geochemistry and can have a significant effect on the chemical profile and that chemical stratification co-occurs with functional gene and taxonomic stratification, it is reasonable to assume that key taxa unique to their respective strata play an integral role in the formation and maintenance of the chemical profile within the sediment column. One likely candidate involved in the sulfate stratification is the Aerophobales bacterium MAG JdF_30, which might significantly contribute to the formation of SMTZs within the observed sediment through the anaerobic oxidation of methane using iron. In the basement-influenced sample D 2H3, the analyzed OGs show a higher potential for denitrification and nitrification than sulfate reduction. As of the time of writing no measurements of nitrate concentrations within sediments from the JdF ridge are available, so only limited conclusions can be drawn. It is, however, conspicuous that the abundance of OG for the Calvin Cycle is even higher than in the upper SMTZ stratum (C 6H6), which is likely a necessary adaptation to deal with the carbohydrate-starved conditions below the secondary SMTZ. This suggests a chemolithoautotrophic lifestyle of the microorganisms living in the sediment, influenced by basement fluid fueled by nitrate reduction rather than sulfate reduction. This is similar to the lifestyle of other bacteria found beneath the great oceanic gyres (Morono et al., 2020). For example, like in sample D 2H3, Chloroflexota and Archaea are nearly absent from the oxic sediment beneath the South Pacific Gyre, while Actinobacteria, Bacteroidetes, Alpha-, Beta-, and Gammaproteobacteria are highly abundant (Morono et al., 2020). It seems, therefore, likely that the anoxic heterotrophy observed in the sulfate-reducing sample C 1H2 is only a useful survival strategy when food in the form of complex carbon molecules is abundant. Archaea and Chloroflexota, which have been suggested to store nutrients obtained from complex and recalcitrant carbon compounds in feast conditions (Hoshino and Inagaki, 2019; Liu et al., 2022), giving them the role of haymakers, only flourish when conditions are sufficient but diminish when they are forced to live in an oligotrophic environment.

In conclusion, the data presented in this study provide new insights into the microbial communities and the metabolic potential of deep sediment layers overlying active ocean ridge flanks. Several MAGs were reconstructed, two of which possibly represent novel taxa on the phylum level. Furthermore, we have uncovered a huge reservoir of “functional dark matter” in the form of uncharacterized protein sequences. These so-called hypothetical proteins might harbor novel and useful traits for applications in biotechnology. Abundance profiling of predicted rRNA genes and marker genes and analysis of OGs for functional genes show that chemical stratification is accompanied by taxonomic and functional stratification. Furthermore, the data show an increase in nitrate reduction-related genes, indicating that nitrate might be available until just below the secondary SMTZ, where microbes use it as a preferred oxidant. The MAGs and the analyses of their metabolic potential are also hinting at a possible role of Aerophobetes in the formation and maintenance of SMTZs. In addition, this study shows evidence that acridine orange staining is not a reliable proxy for live cell counts in deeper sediment layers. The way cell counts are determined in such layers and the weighting of such estimates in ecological studies should, therefore, be reconsidered. Finally, the presented method could be applied to other deep-sea sediments to enable the extraction of sufficient amounts of DNA for metagenomics from relatively low sample volumes, which could enable researchers to additionally apply complementary methods such as single-cell genomics to gain even more detailed pictures of these habitats.

## Data availability statement

The datasets presented in this study can be found in online repositories. The names of the repository/repositories and accession number(s) can be found in the article/Supplementary material.

## Author contributions

FM conducted the experiments. FM, JV, and FL conducted the bioinformatics analyses. JV, FL, A-KK and FM wrote the manuscript. A-KK acquired the funding. All authors contributed to the article and approved the submitted version.
