# Supplementary material for: First shotgun metagenomics study of Juan de Fuca deep-sea sediments reveals distinct microbial communities above, within, between, and below sulfate methane transition zones
Source: Front Microbiol. 2023 Nov 20;14:1241810. doi: 10.3389/fmicb.2023.1241810 (PMC10694467; doi:10.3389/fmicb.2023.1241810)
Supplement: Supplementary file 6 [file Data_Sheet_1.DOCX]

1. Distribute 5 grams of sediment over ten different 15 ml Falcons (0.5 gram per tube) (make sure to use freeze-thaw resistant tubes).
2. Suspend the sediment in 3.7ml per gram 0.1mM EDTA in 1xPBS.
3. Incubate the Falcons 15min at 60°C while shaking at 180rpm.
4. Centrifuge 15 min at 5000rpm.
5. Remove supernatant, re-suspend the sediment in 978µl sodium phosphate buffer.
6. Freeze thaw by submerging the tube in liquid nitrogen until fully frozen and thawing it in a water bath at 60°C.
7. Repeat the freeze-thaw two more times.
8. Transfer the suspended soil to a lysing matrix E.
9. Add 122µl MT-buffer. Vortex briefly to mix.
10. Place lysing matrices in 60°C water bath for 20 minutes.
11. Bead beat the tubes three times for 30 seconds at 6m/s with 30 second breaks in between.
12. Centrifuge 15 minutes at 14000g.
13. Transfer supernatant to clean 1.5ml tubes.
14. Add 250µl PPS.
15. Place on a rotator and rotate the tubes 10 times.
16. Centrifuge 5 minutes at 14000g.
17. Transfer the supernatant to a 15ml falcon, pooling the content from 5 lysing matrices into one falcon.
18. Add 5 ml re-suspended binding matrix (1ml per lysing matrix put in).
19. Place on rotator, avoiding settling of the matrix, and rotate 1h at 25rpm.
20. Remove the falcons and let them sit for 30 minutes to settle matrix.
